# The Dutch Yips Study: Results of a Survey Among Golfers

**DOI:** 10.5334/tohm.636

**Published:** 2021-07-08

**Authors:** Erik van Wensen, Hester J. van der Zaag-Loonen, Bart P. van de Warrenburg

**Affiliations:** 1 Gelre Hospitals, A. Schweitzerlaan 31, 7334 DZ, Apeldoorn, the Netherlands; 2 Sports Dystonia Centre, Laan van Westenenk 4, 7336 AZ, Apeldoorn, the Netherlands; 3 University Medical Center Groningen, Hanzeplein 1, 9713 GZ, Groningen, the Netherlands; 4 Radboud University Medical Center, Reinier Postlaan 4, 6525 GC, Nijmegen, the Netherlands; 5 Department of Neurology, Donders Institute for Brain, Cognition and Behaviour, Montessorilaan 3, 6525 HR, Nijmegen, the Netherlands

**Keywords:** yips, golf, task-specific dystonia, prevalence, sports

## Abstract

**Background:**

The yips in golf is currently regarded as a task-specific movement disorder, with variable phenomenology and of unclear etiology. There is some overlap with task-specific dystonia (TSD), which has also been reported in other sports. The objective was to further characterize the yips in terms of its prevalence and related factors.

**Methods:**

Recreational golfers from one of the larger golf clubs in the Netherlands aged 18 years or older, filled in an anonymous, web-based questionnaire with items on demographic, medical and lifestyle factors, specific yips-relevant items, as well as fanaticism, familial presence of yips, obsessive-compulsive traits, and a dystonia questionnaire.

**Results:**

In total, 234 golfers (26%) completed the questionnaire, among whom 52 (22%, 95% CI: 17–28%) reported to suffer from the yips. In comparison to their non-yips counterparts, the yips group was characterized by a larger proportion of men, more current or past smoking, better golf skills, longer history of playing golf, and more familial yips occurrence.

**Discussion:**

Golfer’s self-reported yips may be very frequent in a group of responding amateur golfers and associated factors seems to include male gender, current or past smoking, extensive golf experience and skills, and a positive family history of the yips. Further work to better understand the origin and nature of the yips is needed.

## Introduction

It has been over 50 years since the first written notion of the yips [1]. The yips is mostly a small, involuntary spasm in the arms while playing golf, especially during a short precise stroke, a “putt”, sometimes producing a complete mishit. Estimated prevalence rates vary between 12% [2] up to almost 50% [3], with most studies targeting selected groups of more experienced golfers.

The golfer’s yips are regarded as a task-specific movement disorders, in part overlapping with or resembling task-specific dystonia (TSD). In terms of phenomenology, the golfer’s yips are, however, not always typically dystonic movements. While electromyography studies have shown muscle co-contraction compatible with dystonia [4], there are also alternative hypotheses on the origin and nature of the yips, as well as studies that distinguish up to five various types of yips, ranging from a jerky type in novices to a dystonic type [5]. In a study by Adler et al [4], only 5 of 14 golfers with the yips were considered to have dystonia.

Like other sport-related movement disorders, such as TSD’s occurring during speedskating, darts, cricket and baseball, the perception is that particularly professional athletes and the better amateurs are affected. The underlying concept is that such TSD’s emerge as a consequence of highly specific, skilled and repetitive movements, in the context of intense training focus, striving for perfection. Sport-related TSD’s can lead to diminished sports-skills, less love of the game and someone having to temporarily interrupt or permanently stop the sporting career.

In an attempt to further understand the yips among a group of normal golfers, in terms of its overall prevalence and factors that might contribute to its emergence, we have conducted a questionnaire study in sample of Dutch recreational golfers.

## Methods

### Study population and study design

We approached the members of a regular large golf club in the Netherlands, the Rosendaelsche Golf Club, to complete a questionnaire. All members (n=912) aged 18 years or older were approached up to 5 repeated mailings and via the homepage of the golf club. Participants anonymously filled in a web-based questionnaire between 1 June and 31 December 2018. A small reward was offered to stimulate participation. Participants could choose from a ticket for a Dutch golf tournament, the KLM-open, or a sleeve of golf balls. We contacted the institutional review board for ethical consent, and received a waiver because of the anonymous nature of the questionnaire.

### Questionnaire items

We asked participants to complete questions about different items (see ***Table 1***). This included, among others, their present and lowest golf handicap (HCP, i.e. a worldwide numeric, downward representation of the skills of a golfer, ranging roughly from 54 (novice) to 0 (expert)). They needed to indicate whether they were suffering from the yips, choosing yes, no, or uncertain. The yips were explained by means of an explanatory video, of which the link was given in the questionnaire (*https://youtu.be/wjV4CFcOflQ*). This video shows the most common form of the yips in the right hand of a victim: a short, jerky, involuntary movement during a putt. Participants who answered “uncertain” were categorized into the “No Yips” (NY) group, to keep the “Yips” (Y) group as pure as possible. Also, they had to fill in a visual analogue scale on fanaticism in sports, on fanaticism in golf, and on the presence of pre-game tension. We added questions targeting possible obsessive-compulsion traits, which were explained within the questionnaire text. Lastly, a Dutch version (not validated) of a dystonia screening questionnaire was administered (see ***Table 1***) [6].

**Table 1:** The Dutch Yips Study Questionnaire.


**1.**	What is your gender?			

**2.**	What is your year of birth?			

**3.**	What is your lowest golf-handicap thus far?			

**4.**	What is your present golf-handicap?			

**5.**	What is your dominant hand?			

**6.**	In what year did you started with playing golf??			

**7.**	Do you have the yips? If no proceed to question 13			

**8.**	When do you have the yips? During: Putting, Chipping, Irons and/or Driver			

**9.**	In what year were you struck by the yips?			

**10.**	In what hand do you have the yips? Right, Left, Both, Do not know			

**11.**	How many units of alcohol do you use in a week? 0, 1–7, 8–14, 14 +			

**12.**	How many cigarettes do you smoke in a week? 0, 1–10, 10 +, stopped			

**13.**	How fanatic are you in sports? 0–10 (0 = I do not care, 10 = most fanatic)			

**14.**	How fanatic are you in golf? 0–10 (0 = I do not care, 10 = most fanatic)			

**15.**	How tense are you before a golf match? 0–10 (0 = not at all, 10 = extreme)			

**16.**	Do you have obsessive thoughts or actions?			

**17.**	Do you use any kind of medication? If yes, please fill in			

**18.**	Are there any kind of neurological disorders in your family?			

**19.**	Do or did you have a neurological disorder?			

**20.**	Is there a first-degree family-member with the yips?			

**21.**	Dystonia Screening Questionnaire	Yes	No	Don’t know

1.	Do you ever close both eyes spontaneously against your own will?			

2.	Do you frequently blink both eyelids against your own will?			

3.	Do you ever open and close your mouth spontaneously against your own will?			

4.	Do your lips ever move spontaneously against your own will?			

5.	Does your tongue ever move spontaneously against your own will?			

6.	Have you ever noticed alterations in voice lowering of voice, choked voice?			

7.	Does your head ever move spontaneously against your own will?			

8.	Do you ever shake your head against your own will?			

9.	Does your arm or hand ever move against your own will?			

10.	Does your arm or hand ever stop moving against your own will?			


### Statistical analysis

We computed prevalence rates and we computed 95% confidence intervals (CI). We used the Chi Square-test and a Students T-test to compare nominal and continuous, for the normally distributed variables between participants with and without the yips, respectively.

### Ethics statement

The approval of an institutional board was not required for this work and patient informed consent was also not required for this work, after consulting an ethical committee.

## Results

Out of 912 eligible golfers, of which 557 (61%) were men, 234 (26%) completed the questionnaire. Among them, 150 (64%) were men and the mean age was 61 years (+SD 12.1). The respondents had been playing golf for a median period of 23 years (interquartile range 16–32), had a mean HCP of 18.6 (+SD 8.2), and their lowest ever HCP was a mean of 16.4 (+SD 8.1) (see ***Table 2***).

**Table 2:** Results of the Dutch Yips Study.


			**P-value**

Participants (234)	Yips = 52 (22%)	Non-Yips = 182 (78%)	

Male gender	45 (87%)	105 (58%)	**<0.001**

Age (mean, SD)	61.7 (± SD 12.5)	60.7 (± SD 12.0)	0.6

Dominant hand			

Right	45 (87%)	157 (86%)	0.91

Left	6 (12%)	22 (12%)	0.91

Ambidextrous	1 (2%)	3 (2%)	NV

Years of Golf	29.5 (+13.9)	23.5 (+11.8)	**0.002**

Present HCP	15.6 (+7.6)	19.3 (+8.2)	**0.002**

Best HCP ever	12.4 (+7.0)	17.5 (+8.0)	**< 0.001**

Smoking (ex)	15 (29%)	24 (13%)	**0.018**

Alcohol > 14 IU p/w	9 (17%)	16 (9%)	0.14

VAS	Mean (+SD)	Mean (+SD)	

VAS-Fanaticism in Golf	7.4 (+1.3)	7.0 (+1.6)	0.1

VAS-Fanaticism in Sports	7.2 (+1.4)	6.8 (+1.5)	0.09

VAS-Competition Tension	6.0 (+2.1)	5.6 (+2.0)	0.2

OCT			

Yes	5 (10%)	11(6%)	0.26

No	38 (73%)	141(77%)	

?	9 (17%)	30(16%)	

Medication	21(40%)	64(35%)	0.3

Beta-blocker	5(9.6%)	6(3%)	0.07

Medical neurological history	5(9.6%)	15(8%)	0.47

Family neurology	8(15%)	24(13%)	0.42

1st degree Yips +	10 (19%)	8 (4%)	**0.0027**

1st degree Yips –	42 (81%)	174 (96%)	

Dystonia Screening Questionnaire	Median	Median	

	0 (0–3)	0 (0–5)	


Legend table 1: HCP = Handicap, ex = former smoker, IU = International Units, p/w = per week, VAS = Visual Analogical Scale, OCT = Obsessive Compulsive Traits, NV = Not Valid because of insufficient numbers.

Fifty-two golfers reported to suffer from the yips, which presents a prevalence of 22% (95% CI: 17–28). The yips emerged after a median of 6 years (interquartile range 4–14), ranging from 0 to 47 years of playing golf. In the yips group, 45 (87%) were men, whereas in the non-yips group 105 (58%) were men (p < 0.001). The yips occurred in the dominant hand in 25 (49%), in the non-dominant hand in 3 (6%), or in both hands in 7 (13%), while the other 16 did not know in which hand they had the yips and one had the yips in the right hand and was ambidextrous. In the yips group, golfers had been playing golf for more years (29.5 years, +SD 13.9) than in the non-yips group (23.5, +SD 11.8; p-value < 0.01). Mean current and lowest HCP was 15.6 +SD 7.6 and 12.4 +SD 7.0, respectively, in the yips group, which was significantly lower compared to the non-yips-group; 19.3 +SD 8.2 and 17.5 +SD 8.0, respectively (both with a p-value < 0.01). Thirty-five (67%) within the yips group had the yips in one specific activity of the game; the vast majority (23 (44%) during putting, a substantial number of 11 (21%) at chipping and only one during driving. Seventeen (33%) had the yips in more than one activity and one in all four activities of the game. Of the golfers with the yips, 29% smoked or had been smoking in the past, compared to 13% without the yips (p = 0.018). There were no group differences for alcohol intake, the various VAS scores, and obsessive-compulsive traits. In the group of golfers with the yips, there were 10 (19%) participants who indicated that they had a first-degree family member who also has the yips, in comparison to 8 (4%) of the 182 without yips (p-value < 0.01).

With regard to medication, we only found a trend for beta-blocker use, which was 5 (9.6%) in the yips group and 6 (3%) in the non-yips group (p = 0.07). There were no relevant differences in own or family history of neurological disease and in our dystonia screening.

## Discussion

Among the golfers who filled in our questionnaire (response rate 26%), we retrieved a yips prevalence of 22% among the participants. Factors possibly associated with the yips were male gender, smoking, and a positive family history for the yips. In addition, golfers with the yips had been playing golf longer and had better skills, in comparison to those without the yips.

Two earlier questionnaire studies targeting the yips reported similarly high or even higher prevalence rates of 12–28% and 32–48%, but – contrary to our study – these two studies were conducted among golf professionals and/or highly skilled amateurs [2,3].

When we extrapolate the prevalence rate of 22% to the total eligible population of this golfclub, and if all non-responders of the included golfclub were not suffering from the yips, the minimal prevalence rate would still be 5.7%. With 400.000 golfers in the Netherlands, of which 68% are men, and with more than 200 golf courses, extrapolation of this minimal prevalence rate suggests that there could be more than 23.000 golfers affected by the yips in the Netherlands. If one would consider the included golfclub to be a representative golfclub for all golfers, this would be a remarkably high prevalence for a movement disorder. Comparing this to for example musician’s dystonia, which has a prevalence of 1% among professional musicians [7], the here retrieved yips prevalence would argue against a TSD as the single etiology underlying the yips.

Still, there are some similarities with regard to some yips characteristics and the risk profile reported here, and those reported for TSD’s. According to our study, there is an overrepresentation of the male sex and a positive family history for the yips, the latter suggesting a genetic susceptibility. This is in line with musician’s dystonia, where the male gender is also more common [8], and with other focal dystonia’s in which up to 25% of patients have an affected family member [9]. Like for other forms of dystonia [10], we found that smoking could be a risk factor for the emergence of the yips, although the biologic underpinning of this possible association is not yet understood and it could be coincidental. In addition, golfers with the yips had been playing golf longer and had better skills, in comparison to those without the yips, which would correspond with the overall hypothesis for the emergence of TSDs. In our study, the yips occurred mostly in the dominant hand (49%), which is also considered to be the most imported hand in putting, comparable with musician’s dystonia [11]. The yips occurred mainly during putting or chipping, but there is also a substantial number of golfers with combined yips.

One of our hypotheses was that golfers with the yips were thought to be more fanatic in sports and in golf and might feel a higher competition tension than the golfers without the yips, but the various VAS scores did not differ significantly between the two groups. We also found no indication for increased rates of obsessive-compulsive traits between the two groups, which is in contrast to the TSD literature [2,12].

One of the two other questionnaire studies eluded to earlier, reported that golfers with the yips had more obsessional thinking, were older and had more golf experience [2]. These last two items were not confirmed by the other study, and in which obsessive thinking was not included in the questionnaire [3].

We acknowledge several limitations of our study. First and most important, our web-based questionnaire is prone to misclassifications and bias, which could affect data interpretation. As we only extracted the subjective presence versus absence of the yips, without expert confirmation, this could lead to an overestimation because of erroneous self-diagnoses. In addition, there could be a bias towards those affected being more inclined to fill in the questionnaire. Also, golfers who were uncertain of having the yips were categorized in the No Yips group. This was done because we did not want to overestimate the total number of golfers with the yips. Secondly, only 26% of golf club members completed the questionnaire. there could be selective reasons for not participating, like feeling ashamed of having the yips or being unaware of having the yips We were not able to collect any data about the non-responders. Thirdly, questions targeting family history were not too detailed and this aspect requires further study. Lastly, we have used self-designed, unvalidated VAS scores for some items we were interested in. These limitations imply that our conclusions should be interpreted as hypothesis generating rather than hypothesis confirmatory.

We conclude from this questionnaire study that the golfer’s yips as a task-specific movement disorder may be frequent in a group of amateur golfers and that the risk profile seems to consist of male gender, current or past smoking, extensive golf experience and skills, and a positive family history of the yips. Further work is needed to better understand the origin and nature of the yips.
